# Evaluation of the Effectiveness of the Iralvex Gel on the Recurrent Aphthous Stomatitis Management

**DOI:** 10.1155/2014/175378

**Published:** 2014-10-14

**Authors:** Heidar Khademi, Pedram Iranmanesh, Ali Moeini, Atefeh Tavangar

**Affiliations:** ^1^Dental Material Research Centre and Department of Oral Medicine, School of Dentistry, Isfahan University of Medical Sciences, Hezar-Jerib Avenue, Isfahan 8174673461, Iran; ^2^Dental Students' Research Center, School of Dentistry, Isfahan University of Medical Sciences, Isfahan 8174673461, Iran; ^3^Department of Epidemiology and Biostatistics, School of Public Health, Tehran University of Medical Sciences, Tehran 1417613151, Iran

## Abstract

*Introduction.* As there is no definitive treatment for the recurrent aphthous stomatitis (RAS), most of the available therapies aim at decreasing pain and discomfort. The aim of this study was to investigate the effectiveness of the Iralvex gel on the RAS management.* Material and Methods.* In this double-blind and placebo-controlled clinical trial study, twenty patients were treated with the Iralvex gel and the other twenty patients were treated with placebo. In every participant complete healing of lesions, pain duration, and intensity were evaluated. Data were analyzed by independent* t*-test and analysis of variance.* Results.* Pain was relieved after 6.10 ± 0.29 days in the Iralvex group in comparison to 8.00 ± 0.33 days in the placebo group (*P* value ≤0.001). Complete remission in the Iralvex group was after 6.80 ± 0.27 days and 10.20 ± 0.42 days in the placebo group (*P* value ≤ 0.001). Furthermore, significant differences in the pain intensity between Iralvex and placebo group measured on days 1, 3, and 5 were obtained (*P* value ≤ 0.01).* Conclusion.* The results of this study show that Iralvex gel is effective and cheap remedy for treatment of RAS without side effects. This trial is registered with IRCT201207253251N3.

## 1. Introduction

Recurrent aphthous stomatitis (RAS) is regarded as one of the most common oral mucosa lesions, affecting 10–60% of the population [1]. These lesions are painful and alter the life quality of patients by difficulty in eating, speaking, and swallowing [2, 3] Considered as a multifactorial disorder, RAS etiology is unknown. Genetic background, stress and excitement, trauma, hormonal state, foods allergy, immune disorders, and presence of helicobacter pillory are among the factors associated with RAS [3–10].

Due to inadequate understanding of RAS etiologic factors, studies failed to introduce a definite treatment [11, 12] or treatment with “long-term” therapeutic goals [13]. Therefore, primary goals of RAS therapy are focused on sign and symptomatic management such as palliation of pain, a reduction in number, size, recurrence and duration of ulcers, and healing promotion [5, 11–13]. Topical anti-inflammatory, antihistaminic, analgesics, anesthetics agents, immunomodulators, immunosuppressants, as well as steroids agents [11, 13] are prescribed based on the severity of RAS [1, 3, 14]. Some of these medications lead to many side effects or undesirable reactions [11] such as osteoporosis, hyperglycemia, hypertension, adrenal suppression, and immune system suppression [3, 15].

A number of medicinal plants, herbal preparations, and herbal drugs are listed as effective agents for controlling signs and symptoms of RAS [2, 5, 11, 12]. Recently, the* Goldaru Herbal Pharmaceutical Laboratories *introduced the Iralvex drug as an antiaphthous herbal agent. The manufacturer has recommended the Iralvex drug as an effective treatment for inflamed and ulcerated conditions of the mouth, gum bleeding, and aphthous stomatitis [16]. Each gram of the Iralvex gel is composed of 170 mg dried rhubarb extract (standardized on the basis of 3.4–4.6 mg of rhein) and 10 mg salicylic acid [16]. The constituents of the rhubarb extract include anthraquinones (emodin, chrysophanol, rheni, physcion, aloe-emodin, and the glycosides of these substances); anthrone; dianthrones (dianthrones of chrysophanol, emodin, aloe-emodin, and glucorhein); heterodianthrones (palmidins A and B); tannins (glucogallin and gallic acid); and starch and calcium oxalate [16, 17]. In an in vitro study by Atai et al. [18], the antibacterial and antifungal effects of some herbal mouth rinses including Persica, Matrica, and Iralvex were compared with Chlorhexidine 0.2%. The results indicated that the herbal mouth rinses were less effective than Chlorhexidine. In a clinical trial, Yazdizadeh et al. [19] compared the effects of the Iralvex gel and the lidocaine gel 2% on RAS lesions. They reported the similar effectiveness of two gels on pain relief, lesion size, and healing time of RAS.

Application of herbal remedies for diseases is highly valuable since herbal drugs are natural and more body compatible compared to chemical medications; therefore, investigations on herbal drugs are recommended. The aim of the present double-blind, placebo-controlled trial study was to investigate the effectiveness of the Iralvex gel on RAS management based on the manufacture claim.

## 2. Method

### 2.1. Ethical Approval

This study was approved by the Medical Research Ethics Committee of Isfahan University of Medical Sciences (Grant no. 391105) and has no conflict with the Declaration of Helsinki.

### 2.2. Patients

Among the outpatients attending, forty patients (14 males and 26 females) who were referred to the Department of Oral Medicine, School of Dentistry, Isfahan University of Medical Sciences, were selected.

### 2.3. Inclusion and Exclusion Criteria

The individuals who had suffered from systemic diseases or immunologic disease or special syndrome concurrent with oral lesions “(like Behcet's disease, rheumatoid arthritis, lupus, acquired immune defi-ciency syndrome, Reiter's disease, bowel disease, celiac disease, major or herpetiform aphthous lesions, erythema multiform, and vesiculobullous diseases) or those with aphthous lesions older than 2 days from initiation or patients who had been receiving other concurrent medication for aphthous ulcers either systematically or locally for at least 4 weeks before the study begins and those without tendency for entering the study were excluded.

The patient's RAS were diagnosed by the patient's medical history, clinical examination, and the presence of a well demarcated painful ulcer with a diameter less than 1 cm in nonkeratinized oral mucosa, which was covered by white removable membrane and surrounded by light red areola [3]. The patients also reported to be affected by minor RAS at least 3 times a year with healing without scars through lesion duration of 10–14 days. Due to the pain associated with the ulcer, all individuals had difficulty in eating meals.

### 2.4. Study Design

The patients were randomly assigned into experimental and placebo groups (20 patients per each group). The products were placed in exactly similar containers and were coded by a third person, so that 20 individuals randomly received the Iralvex gel package (15 g tubes) and the other 20 received the placebo gel. The placebo gel contained ferric oxide. Following the instructions of the Iralvex gel's manufacturer, the patients were asked to apply 1 cm of the gel on their ulcers three times a day by using a small sterile cotton pad impregnated and rub it gently [16]. The patients were supposed to refrain from eating and drinking for at least 30 minutes after drug application.

After receiving instructions, the patients were asked to record the aphthous pain level on days 0, 1, 3, and 5 based on visual analogue scale (VAS) using a number from 0 to 10. Moreover, they were informed to record the date of pain elimination and complete remission. Complete healing was confirmed by clinician examination. The time when the pseudomembrane and the erythematous border were removed was regarded as the complete healing date. During the treatment, researchers were constantly in contact with the patients to ensure they were following the instructions, recording the results correctly, and determining any adverse reactions.

After 1 week, the patients were examined again and the questionnaires were collected. At the end of the study the tubes were decoded.

### 2.5. Statistical Analysis

The statistical analyses were conducted by SPSS16 software using repeated measure ANOVA and independent *t*-test. *P*  value < 0.05 was considered statistically significant.

## 3. Results

Out of 40 patients, 14 were males and 26 were females (7 males and 13 females in each group). The mean ages of the participants in the study and control groups were 25.80 ± 1.53 and 26.20 ± 1.64, respectively. There were no significant differences between the two groups in terms of gender and age.

**Figure 1:**
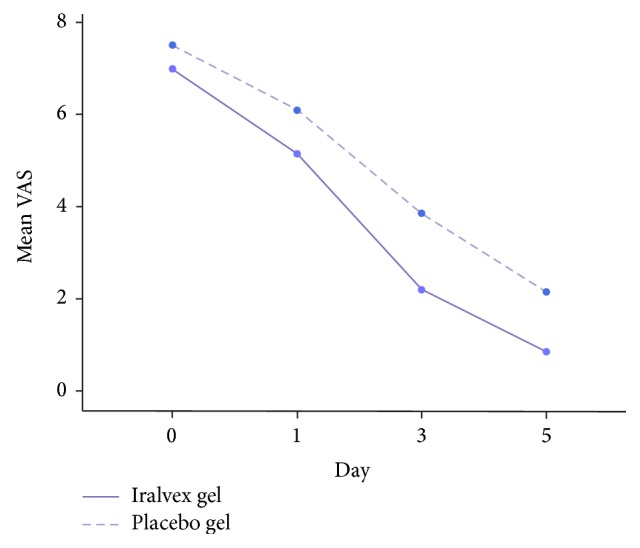
The mean severity of pain for the Iralvex and placebo groups in different days according to visual analogue scale.

Twenty patients (50%) received the Iralvex gel as the treatment group and 20 patients (50%) received placebo as the control group. Although the mean VAS scores decreased from the onset of product usage to day 5 in both groups, the mean VAS scores in the placebo group were higher than the treatment group (repeated measure, *P*  value ≤ 0.001). The differences in the pain level on 0 day were not statistically significant; however the mean VAS scores on days 1, 3, and 5 were significantly lower in the Iralvex group (independent *t*-test, *P*  value ≤ 0.01) (Table 1 and Figure 1).

The mean pain durations in the experimental and placebo groups were 6.10 ± 0.29 and 8.00 ± 0.33 days, respectively. Statistical analysis found a significant difference between the two groups in this regard (independent *t*-test, *P*  value ≤ 0.001). Furthermore, the time of complete healing of RAS lesions was recorded. The mean in the treatment and control group was 6.80 ± 0.27 and 10.20 ± 0.42 days, respectively (independent *t*-test, *P*  value ≤ 0.001). Additionally, no patient reported any side effects from the Iralvex gel as medication.

Moreover, medical history of patients was determined by self-assessed questionnaire. The result showed that 90% of the patients had a history of stress, 75% of the patients had a history of food allergy, as many as 60% of individuals had a trauma history, and 10% used to smoke before the presence of ulcers.

## 4. Discussion

RAS is considered as a common oral disease, and, due to uncertain etiology, no definitive treatment has been reported for this oral lesion [3, 20]. Pain is the most frequent complaint of patients with ulceration [21]. However, by suitable pain control, complicated treatments are avoidable [12]. Various herbal preparations have been reported for RAS management including* Myrtus communis* [11],* chamomilla* [2],* Satureja khuzestanica* [12],* Zataria multiflora*,* Anthemis nobilis* [11], and* Punica granatum* [21].

The Iralvex gel is a herbal drug introduced for RAS management. The drug contains 170 mg dried rhubarb extract and 10 mg salicylic acid [16]. A few studies investigated the effect of the Iralvex gel on RAS management. In a clinical trial study, similar effectiveness of the Iralvex gel and the lidocaine gel on pain relief, lesion size, and healing time of RAS was reported [19]. In the present study, the Iralvex gel showed a significant effectiveness on RAS management. While there was no significant difference between the two groups in terms of the mean pain level at 0 day, the mean was significantly lower in the Iralvex group at 1, 3, and 5 days. Furthermore, the mean duration of pain in the treatment group (6 days) was significantly lower than the other group (8 days). The complete healing time was 7 days in the treatment group and was significantly lower than the control group (10 days). The ingredients of the Iralvex may have contributed to the alleviation of pain and faster wound healing. Anthraquinone glycosides, tannin, and salicylic acid in the product react with the proteins of the epithelial cells in the mucous membranes and lead to a stronger adhesion of the mucosa and a reduction in the permeability of cells. This process is known as astringency which protects the outer layer of gingival mucosa against the microbes and toxin [17]. Moreover, antibacteria activity these chemicals could have inhibited the growth of some bacteria including* Staphylococcus aureus*,* Proteus*,* Neisseria*,* and Candida albicans*, leading to better pain alleviation and earlier wound healing [17].

Jafari et al. [11] compared the healing effects of* Myrtus Communis*,* Zataria multiflora*,* Anthemis nobilis*, and mixture of* Zataria multiflora* and* Anthemis nobilis* on minor RAS. The mean date of complete healing (7 days) and complete pain relief (6 days) of the Iralvex gel was in accordance with the results of Myrtle. Moreover, the Myrtle and the Iralvex gel both contained tannin compounds which are likely to provoke astringency and protect the outer layer of gingival mucosa against the microbes and stimulus chemicals. Therefore, the tannin may be considered as the main reason for RAS management in the Iralvex gel.

Stress and excitement [6, 22], allergy [3, 9] trauma [3, 20], and smoking cessation [3, 23] have been reported as contributing factors of RAS. Although psychological stress is reported to be associated with 60% of the first presence of aphthous ulcers and 20% of RAS [24], it may act as a trigger factor rather than an etiological factor [6]. Food allergies like chocolate, cheese, tomato, strawberry, wheat flour, and peanut are also reported as other associated factors of RAS [3, 24]. Furthermore, aphthous ulcers are more likely to occur in traumatic mucous [3, 20]. Most of RAS' patients are nonsmokers and, in some individuals, aphthous ulcers occur after quitting smoking and disappear after resmoking. This protective effect of smoking on RAS was dose- and time-dependent [23]. The reason may be the positive effect of nicotine and tobacco on keratinization of oral epithelial [23, 24]. In the present study, the patients were reported to have a history of stress (90%), food allergy (75%), trauma (60%), and smoking (10%) before the presence of ulcers. Therefore, they may be considered as predisposing factors of aphthous lesions. However, more studies are necessary on the contributing factors of RAS.

The short-term follow-ups are considered as the limitations of the present study. Further studies should be undertaken to investigate the effect of the Iralvex gel on the recurrence of aphthous ulcers with long-term follow-ups. In the present study, medical history of patients was determined by self-assessed questionnaire; it is recommended in future studies that the medical history of patients be approved by clinical examination. Although there is no standard drug for RAS, more studies with a larger number of patients for comparing the effectiveness of the Iralvex gel with other usual medicines (as active control) are recommended.

## 5. Conclusion

The present study investigated the effectiveness of the Iralvex gel on RAS management. Within the limitation of this study, the following conclusions could be proved.

1. As an inexpensive herbal drug, the Iralvex gel is an effective remedy for both pain relief and healing of aphthous ulcers with no noticeable side effect.
2. Stress, food allergy, and trauma are the factors associated with RAS.

## Figures and Tables

**Table 1:** Comparison of VAS score for the Iralvex and placebo groups in different days.

Group	VAS mean ± SE			
	Day 0	Day 1	Day 3	Day 5
Iralvex gel	7.00 ± 0.26	5.15 ± 0.21	2.20 ± 0.16	0.85 ± 0.17
Placebo gel	7.50 ± 0.29	6.10 ± 0.25	3.85 ± 0.24	2.15 ± 0.22

*P* value^†^	0.205	<0.01∗	<0.001∗	<0.001∗

VAS: visual analogue scale.

∗Significant association set at 5.0%.

^†^Independent *t*-test.
